# Magnetic Resonance Imaging for Quantitative Assessment of Lung Aeration: A Pilot Translational Study

**DOI:** 10.3389/fphys.2018.01120

**Published:** 2018-08-13

**Authors:** Lorenzo Ball, Anja Braune, Peter Spieth, Moritz Herzog, Karthikka Chandrapatham, Volker Hietschold, Marcus J. Schultz, Nicolò Patroniti, Paolo Pelosi, Marcelo Gama de Abreu

**Affiliations:** ^1^Department of Anesthesiology and Intensive Care Medicine, Pulmonary Engineering Group, University Hospital Carl Gustav Carus, Technische Universität Dresden, Dresden, Germany; ^2^Department of Surgical Sciences and Integrated Diagnostics, Università degli Studi di Genova, Genoa, Italy; ^3^Ospedale Policlinico San Martino, Genoa, Italy; ^4^Department of Intensive Care, Academic Medical Center, University of Amsterdam, Amsterdam, Netherlands; ^5^Department of Radiology, University Hospital Carl Gustav Carus, Technische Universität Dresden, Dresden, Germany

**Keywords:** lung, magnetic resonance, aeration, *ex vivo* model, atelectasis

## Abstract

**Background:** Computed tomography is the gold standard for lung aeration assessment, but exposure to ionizing radiation limits its application. We assessed the ability of magnetic resonance imaging (MRI) to detect changes in lung aeration in *ex vivo* isolated swine lung and the potential of translation of the findings to human MRI scans.

**Methods:** We performed MRI scans in 11 isolated non-injured and injured swine lungs, as well as 6 patients both pre- and post-operatively. Images were obtained using a 1.5 T MRI scanner, with T_1_ – weighted volumetric interpolated breath-hold examination (VIBE) and T_2_ – weighted half-Fourier acquisition single-shot turbo spin-echo (HASTE) sequences. We scanned swine lungs, with reference samples of water and muscle, at different airway pressure levels: 0, 40, 10, 2 cmH_2_O. We investigated the relations between MRI signal intensity and both lung density and gas content fraction. We analyzed patients’ images according to the findings of the *ex vivo* model.

**Results:** In the *ex vivo* samples, the lung T_1_ – VIBE signal intensity normalized to water or muscle reference signal correlated with lung density (*r*^2^ = 0.98). Thresholds for poorly and non-aerated lung tissue, expressed as MRI intensity attenuation factor compared to the deflated lung, were estimated as 0.70 [95% CI: 0.65–0.74] and 0.28 [95% CI: 0.27–0.30], respectively. In patients, dorsal versus ventral regions had a higher MRI signal intensity both pre- and post-operatively (*p* = 0.031). Comparing post- versus pre-operative scans, lung volume decreased (*p* = 0.028), while the following increased: MRI signal intensity in ventral (*p* = 0.043) and dorsal (*p* < 0.0001) regions, and percentages of non-aerated (*p* = 0.028) and poorly aerated tissue volumes (*p* = 0.028).

**Conclusion:** Magnetic resonance imaging signal intensity is a function of lung density, decreasing linearly with increasing gas content. Lung MRI might be useful for estimating lung aeration. Compared to CT, this technique is radiation-free but requires a longer acquisition time and has a lower spatial resolution.

## Introduction

The quantitative analysis of lung aeration, usually performed with computed tomography (CT), has deeply changed the understanding and clinical management of the acute respiratory distress syndrome (ARDS) (Puybasset et al., 1998; Gattinoni et al., 2001). Several imaging tools without ionizing radiation exposure have been proposed to estimate lung aeration, including lung ultrasound and electrical impedance tomography (Ball et al., 2013). Safety concerns limit the clinical application of quantitative lung CT to critically ill patients, where the high mortality and the clinical value of this technique possibly justify the biological hazards of radiation exposure.

Over the last decade, faster magnetic resonance imaging (MRI) sequences have made available, capable of acquiring vast portions of the chest in a single breath-hold, thus expanding the clinical applications of lung MRI (Abolmaali et al., 2004; Biederer et al., 2012). Differently from CT, MRI signal intensity is not calibrated to a standard measurement unit, making quantitative analysis of images more difficult. The presence of air in lungs, reducing the number of signal-generating protons to around one fifth of the average proton density of other organs, leads to a low MRI signal intensity (Mulkern et al., 2014). Despite this, a ventro-dorsal gradient in lung MRI signal consistent with the gravitational water displacement in the supine position has been described (Mulkern et al., 2014; Pusterla et al., 2016).

The objective of the present study was to assess the ability of T_1_ and T_2_-weighted sequences to detect differences in lung aeration in an *ex vivo*, isolated swine model of healthy and injured lung. Also, we aimed at determining the potential of translation of the method to clinical MRI scans. We hypothesized that MRI scans can be used for assessment of quantitative lung aeration.

## Methods

### *Ex vivo* Lung Samples Preparation

We obtained lungs and *erector spinae* muscles of nine adult pigs from a slaughterhouse in Dresden, Germany. The *ex vivo* study involved animal samples discarded from the slaughter, therefore no ethical approval was required according to national regulations. The lung preparation procedure was adapted from a previous study of our group (Corradi et al., 2013). Briefly, immediately after slaughtering we extracted lung-trachea blocks from the animals and transported the blocks to our research facility within 2 h. We discarded 7 blocks due to parenchymal lesions, yielding 11 intact lungs, which were then intubated endobronchially (∅ 6 mm, Rüsch, Kernen, Germany). In 3 lungs, we instilled 0.5 mL of 0.45% sodium chloride solution per gram of lung tissue inside the bronchi, to mimic extravascular lung water (EVLW) (injured samples). In the remaining 8 lungs, no instillation was performed (non-injured samples). Blocks were stored for 10 h at 4°C, to promote complete deflation and fluids distribution within the lung parenchyma in the injured samples. Afterwards, excess of fluids was gently suctioned through the endobronchial tubes.

### Image Acquisition Protocol

We connected the lung samples to an MRI-compatible mechanical ventilator (Fabius MRI, Dräger, Germany) set in manual mode, and applied a continuous positive airway pressure (CPAP) through the airway pressure-limiting valve with 0.21 fraction of inspired oxygen (FiO_2_). We performed MRI scans (see details below) initially at 0 cmH_2_O. Following a recruitment maneuver with CPAP of 50 cmH_2_O for 30 s, we performed scans sequentially at 40, 10, and 2 cmH_2_O CPAP. Two sealed polypropylene tubes containing 50 mL of distilled water, or 50 ± 5 g of muscle tissue served as reference.

We used a MAGNETOM Avanto 1.5 Tesla scanner (Siemens, Erlangen, Germany) using two different MRI sequences: T_1_-weighted gradient-echo volumetric interpolated breath-hold examination (T_1_ – VIBE, TR = 5 ms, TE = 2 ms, acquisition time 12 s per 15 cm volume block, 4 mm slice thickness, 1.37 mm × 1.37 mm pixel size, 256 × 208 voxel matrixes) and T_2_-weighted half-Fourier acquisition single-shot turbo spin-echo (T_2_ – HASTE, TR = 1000 ms, TE = 98 ms, acquisition time 360 ms per slice, 5 mm slice thickness, 1.09 mm × 1.09 mm voxel size, 320× 244 voxel matrixes). We performed the *ex vivo* scans on three distinct days, immediately after the self-calibration procedure of the scanner. Images were retrieved as uncompressed DICOM files, manually segmented with the ITK Snap software package^1^ excluding large airways up to the first bronchial division, and analyzed with a custom-made Matlab script (MathWorks, Natick, MA, United States) to obtain volume and average MRI signal of each sample, expressed in grey level units (GU). After MRI scans, we removed surgically the large airways up to the first bronchial division, to match the MRI segmentation, and we weighted the lung tissue with a precision scale (Acculab ATL 2202, Sartorius Group, Göttingen, Germany). Lung density at each CPAP level was calculated as Density_LUNG_ = Mass_SCALE_/Volume_MRI_ and expressed in g/mL.

### *In vivo* Human Data Sets for Validation

We obtained pre- and post-operative MRI scans of 6 patients included in a non-related trial (NCT01683578), (Spieth et al., 2017) who developed relevant degrees of atelectasis after major abdominal surgery. In that study, patients were ventilated intraoperatively with variable or conventional volume-controlled ventilation, and the post-operative scans were performed in postoperative day 1 to assess the presence of post-operative atelectasis, during spontaneous breathing in supine position. The review board of the Medical Faculty and the University Hospital Dresden, Germany approved the protocol of the clinical study (EK174052011), and written informed consent was obtained from all patients. Scans were performed during inspiratory breath-hold and merging image blocks acquired during two or three consecutive periods of apnea to obtain whole-thorax acquisition, with T_1_ – VIBE sequences as described for the *ex vivo* model.

### Analysis Plan

The primary end-point of the study was the correlation between lung density and MRI signal intensity. We expected a correlation with |*r*|≥ 0.8. Therefore, we needed to include at least 9 lung samples to achieve 90% power (1–β) to detect such correlation with α < 0.05.

Water, muscle and air signal intensity was calculated as average intensity of a spherical region of interest (ROI) of 2.03 cm^3^. MRI signal was either expressed in raw GU, or normalized dividing by the signal intensity of the water or the muscle sample. For each lung, the MRI signal (MRI_0_) and lung volume (V_0_) measured with the sample at its minimum inflation level at zero airway pressure were used as reference. We then calculated the relative change in volume and MRI signal at increasing airway pressure. Lung MRI signal intensity attenuation at each CPAP level was defined as MRI_ATT_ = MRI_CPAP_/MRI_0_, and gas fraction as GAS_F_ = (V_CPAP_–V_0_)/V_CPAP_. MRI_ATT_ was plotted and fitted against GAS_F_, to estimate thresholds separating poorly and non-aerated lung areas. GAS_F_ cut-off values were set as in CT studies: (Gattinoni et al., 1988) GAS_F_ < 0.1 was defined as non-aerated lung tissue, 0.1 < GAS_F_ < 0.5 poorly aerated, while GAS_F_ ≥ 0.5 was considered as normal aeration or hyperaeration. The signal-to-noise ratio was calculated as SNR = MRI_LUNG_/MRI_AIR_.

In human scans, we sampled the MRI signal intensity of a muscle in the middle axial slice of each MRI acquisition block, averaging signal from a ROI of 0.46 cm^2^, and all MRI intensity values were normalized dividing by this value. A reference for intensity of a non-aerated portion of the lung (MRI_ATEL_) was obtained averaging the lung signal arising from a cubic ROI of 2.03 cm^3^ placed in the middle of the non-aerated tissue. Thresholds for non-aeration and poor aeration in the *in vivo* scans were calculated as MRI_ATEL_ × MRI_ATT, THRESHOLD_. Aeration was assessed in the whole lung and in two equally spaced ventro-dorsal ROIs.

Non-linear mixed effects models were used to investigate the relationship between Density_LUNG_ and MRI signal intensity and between GAS_F_ and MRI_ATT_. The mixed model included a term to account for repeated measurements in the same sample, and a dichotomous variable to identify healthy or injured lungs. Statistical models were compared with the corrected Akaike information criterion (AICc), testing linear (*y* = a.x+b) and quadratic (*y* = a.x^2^+b) functions with a heuristic approach. In patients’ scans, differences between the pre- and post-operative scans were tested with a 2-way ANOVA for repeated measurements with Sidak *post hoc*, while comparisons between quantitative analysis parameters before and after surgery were sought with Wilcoxon test. Data are reported as mean ± standard deviation or as median (25th to 75th percentiles), as appropriate. Statistical analysis was performed with R version 3.2.3 ^2^ and significance assumed for *p* < 0.05.

## Results

Porcine lung samples had a mass of 131.4 ± 34.6 g, and the injured samples retained 0.33 ± 0.02 g of EVLW per gram of lung tissue. The characteristics of the samples are reported in **Table 1**.

**Table 1:** Lung samples characteristics.

Sample no.	Group	Side	Pre-instillation (g)	Post-instillation (g)	Without airways (g)	% EVLW
1	Healthy	Left	113.0	–	93.6	–
2	”	Right	169.9	–	123.0	–
3	”	Left	139.9	–	120.3	–
4	”	Right	197.4	–	152.4	–
5	”	Left	222.2	–	183.4	–
6	”	Right	141.7	–	114.1	–
7	”	Right	118.2	–	92.7	–
8	”	Left	96.2	–	77.7	–
9	Injured	Right	165.8	223.4	185.6	31%
10	”	Left	134.1	183.8	152.5	33%
11	”	Left	172.6	226.1	149.7	36%

*Lung mass is reported before and after instillation for injured lungs. Mass without airways refers to the mass of the lung sample after removal of the endotracheal tube and surgical excision of the largest airways up to the first bronchial division. EVLW, Extra Vascular Lung Water, as percent of the lung tissue mass.*

### Signal-to-Noise Ratio and Feasibility of Image Segmentation

The SNR at 0, 2, 10, and 40 cm H_2_O CPAP was, respectively, 58.0 ± 28.2, 22.4 ± 7.1, 14.5 ± 4.2, and 11.1 ± 5.0 in T_1_ – VIBE scans, while 27.8 ± 9.6, 15.4 ± 6.7, 11.6 ± 5.4, and 9.9 ± 5.2 in T_2_ – HASTE scans. Despite the lower SNR achieved with higher CPAP levels, the manual segmentation could be manually performed in all scans, as the interface between lung and air could be identified by visual inspection.

### Relationship Between Lung Density and MRI Signal

**FIGURE 1:**
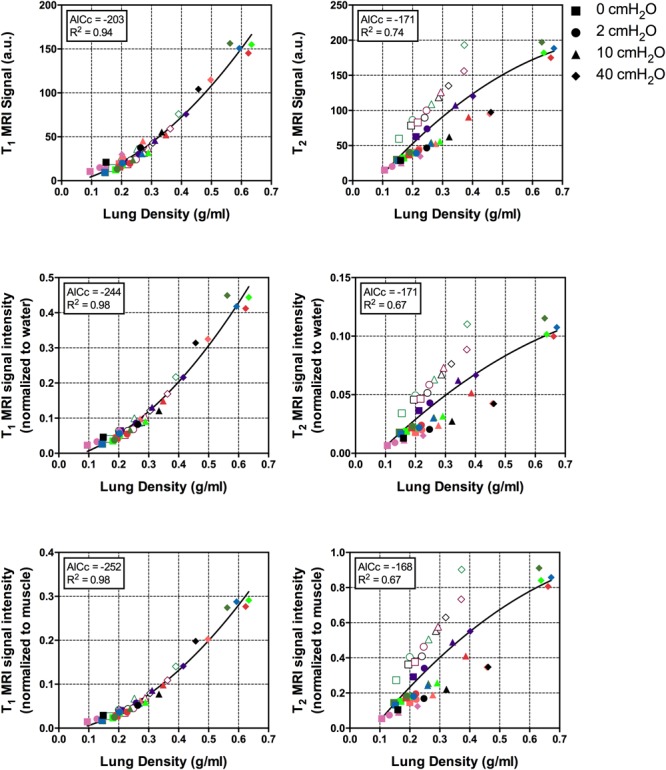
Relationship between lung density and MRI signal intensity, with and without normalization to water or muscle signal. Symbol shapes refer to inflation pressure, as illustrated in the legend, colors indicate the different lung samples; empty symbols: injured samples; filled symbols: healthy samples. AICc, corrected Akaike information criterion; MRI, magnetic resonance imaging.

**FIGURE 2:**
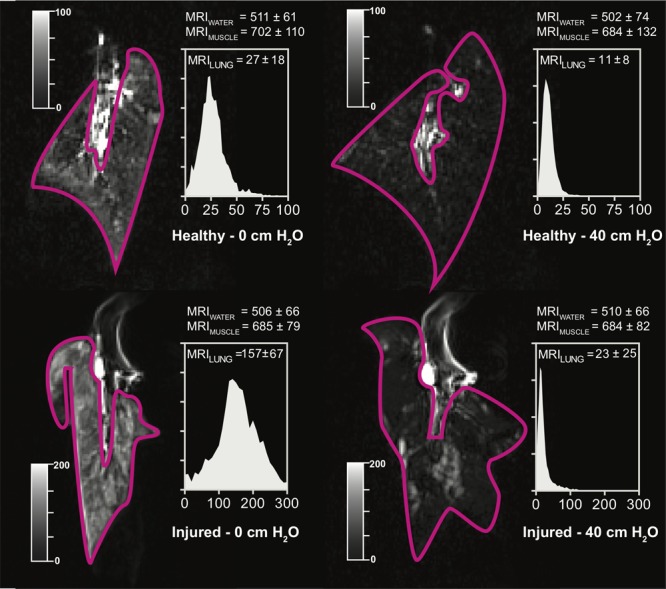
Representative scans obtained with T_1_ – VIBE sequences, in a representative healthy (Top) or injured (Bottom) sample, deflated (Left) and at 40 cm H_2_O CPAP. Image windows was adjusted to optimize lung visualization (see grayscale sliders). Histograms represent the lung voxel intensity frequency distribution; *X*-axis: MRI signal intensity in gray units; *Y*-axis: relative voxel frequency. MRI, magnetic resonance imaging; CPAP, continuous positive airway pressure.

**FIGURE 3:**
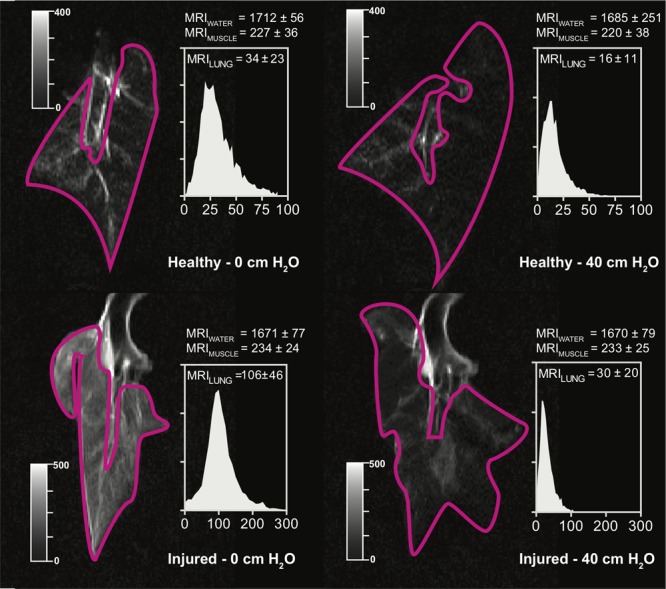
Representative scans obtained with T_2_ – HASTE sequences, in a representative healthy (Top) or injured (Bottom) sample, deflated (Left) and at 40 cm H_2_O CPAP. Image windows was adjusted to optimize lung visualization (see grayscale sliders). Histograms represent the lung voxel intensity frequency distribution; *X*-axis: MRI signal intensity in gray units; *Y*-axis: relative voxel frequency. MRI, magnetic resonance imaging; CPAP, continuous positive airway pressure.

Lung density could be described as a function of MRI signal; as shown in **Table 2**, the lowest AICc values were obtained with the T_1_ – VIBE sequences, MRI normalization to either water or muscle signal, and a quadratic model. In all the models based on T_2_ – HASTE sequences, a significant effect of EVLW on the density-signal relationship was observed (*p* < 0.001). The presence of EVLW did not affect the accuracy of quadratic models based on T_1_ – VIBE sequences and MRI normalization to water (*p* = 0.83) or muscle (*p* = 0.47) signal, and quadratic models performed better than linear models (see **Table 2**). **Figure 1** illustrates the relationship between lung density and MRI signal with or without normalization. Representative MRI scans of healthy and injured samples scanned with T_1_ – VIBE and T_2_ – HASTE sequences are illustrated in **Figures 2** and **3**, respectively.

**Table 2:** AICc for models describing lung density as a function of lung MRI signal intensity, with and without normalization to water and muscle signal.

MRI sequence	Model	Lung MRI signal intensity, absolute	Lung MRI signal intensity, normalized to water	Lung MRI signal intensity, normalized to muscle
T_1_ – VIBE	Linear	–132 (*p* = 0.09)	–158 (*p* = 0.16)	–163 (*p* = 0.13)
	Quadratic	–203 (*p* = 0.28)	–244 (*p* = 0.83)	–252^∗^ (*p* = 0.47)
T_2_ – HASTE	Linear	–137 (*p* < 0.001)^†^	–132 (*p* < 0.001)^†^	–130 (*p* < 0.001)^†^
	Quadratic	–171 (*p* < 0.001)^†^	–171 (*p* < 0.001)^†^	–168 (*p* < 0.001)^†^
				*AICc (p-value for EVLW effect)*

*AICc is expressed in the smaller-is-better form. ^†^Model with a significant effect of the presence of EVLW (p < 0.05); ^∗^Model with lowest, thus better, AICc value. EVLW, extra-vascular lung water; MRI, magnetic resonance imaging; AICc, corrected Akaike information criterion; T_*1*_ – VIBE: T_*1*_ – weighted gradient-echo volumetric interpolated breath-hold examination; T_*2*_ – HASTE: T_*2*_ – weighted half-Fourier acquisition single-shot turbo spin-echo.*

### Relationship Between Lung Gas Content and MRI Signal Attenuation

**FIGURE 4:**
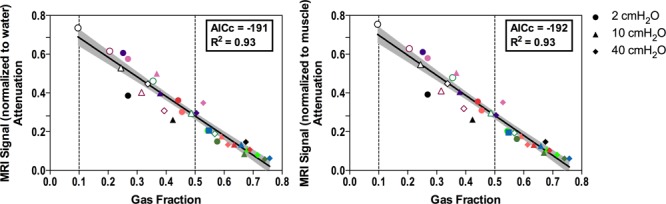
Relationship between lung gas content (Gas Fraction) and MRI signal attenuation compared to the deflated lung. MRI signal is normalized to water **(Left)** or muscle **(Right)**. Dashed lines represent thresholds for non-aerated and poorly aerated lung tissue. Symbol shapes refer to inflation pressure, as illustrated in the legend, colors indicate the different lung samples; empty symbols: injured samples; filled symbols: healthy samples. MRI, magnetic resonance imaging; AICc, corrected Akaike information criterion.

At increasing GAS_F_, MRI signal attenuation compared to the deflated lung sample (MRI_ATT_) decreased linearly, without relevant differences between MRI normalized to water or muscle signal (R^2^ = 0.93 in both cases, **Figure 4**). As shown in **Figure 4**, at the threshold for non-aeration, MRI_ATT_ was 0.70 [95% confidence interval, CI: 0.65–0.74] while the threshold for poorly aerated tissue corresponded to MRI_ATT_ of 0.28 [95% CI: 0.27–0.30].

### Validation in Human Scans

**FIGURE 5:**
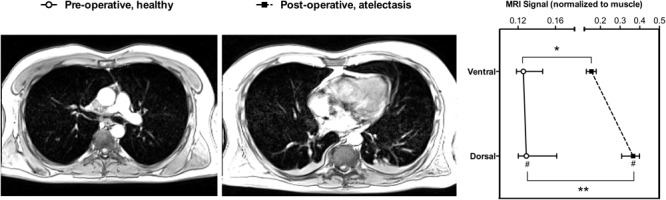
Magnetic resonance imaging quantitative analysis in a representative patient. Pre-operative scan with healthy lungs **(Left)**, and post-operative scan showing atelectasis **(Middle)**. **(Right)** Shows the lung MRI signal intensity, normalized to muscle signal, of two equally spaced ventro-dorsal ROIs in the pre- and post-operative scans. ^#^Higher compared to the ventral ROI (*p* = 0.031); Significantly higher in the post-compared to the pre-operative scan in the corresponding ROI ^∗^*p* = 0.043, ^∗∗^*p* < 0.0001. MRI, magnetic Resonance Imaging; ROI, region of interest. Symbols are median, error bars the interquartile range.

*In vivo*, the acquisition of each 15-cm thorax block required 12 s, and two to three blocks were merged to obtain whole chest images. In patients’ scans, MRI_ATEL_ was 0.92 ± 0.15, therefore the thresholds for non-aerated and poorly aerated lung tissue were set to 0.64 and 0.26, respectively. Variation of these thresholds by ± 5% yielded a negligible absolute variation in lung aeration compartment size estimation (see **Supplementary Figure 1**). Patients’ characteristics and lung MRI quantitative analysis results are illustrated in **Tables 3** and **4**, respectively. **Figure 5** shows MRI scans of a representative patient with healthy lung and with atelectasis, and the MRI signal intensity in the two ventro-dorsal ROIs.

**Table 3:** Patients characteristics.

Variable	
*N*	6
Age	75 [62–80]
Sex (males)	3 (50%)
BMI (kg/m^2^)	26.9 [23.3–28.6]
ASA class	III [II–III]
ARISCAT score	41 [40–59]
Upper abdomen incision	5 (83%)
Lower abdomen incision	1 (17%)
Preoperative SpO_2_ (%) in room air	96 [94–97]
Postoperative SpO_2_ (%) in room air	89 [88–90]
Postoperative FEV_1_ (as % expected)	36 [32–44]
Postoperative FVC (as % expected)	48 [36–102]

*Data are medians [25th–75th quartile] or frequency (proportion). BMI, body mass index; ASA, American Society of Anesthesiologists; ARISCAT, Assess Respiratory Risk in Surgical Patients in Catalonia; FEV_*1*_, forced expiratory volume at 1 s; FVC, forced vital capacity.*

**Table 4:** Results of the quantitative MRI analysis of T_1_ – VIBE scans in humans.

	Pre-operative MRI	Post-operative MRI	*p*
Total lung volume (mL)	4233 [2855-5007]	2464 [1889-2984]	0.028ˆ*
MRI signal (absolute, gray units)	20.4 [18.6-25.1]	36.2 [33.3-45.7]	0.028ˆ*
MRI signal normalized to muscle	0.11 [0.10-0.13]	0.23 [0.20-0.26]	0.028ˆ*
Non-aerated lung volume (mL)	14 [4-20]	245 [188-272]	0.028ˆ*
Non-aerated lung volume (% of TLV)	0.3 [0.1-0.7]	10.1 [8.3-11.1]	0.028ˆ*
Poorly aerated lung volume (ml)	318 [246-453]	403 [354-438]	0.35
Poorly aerated lung volume (% of TLV)	7.7 [5.9-12.0]	16.0 [13.3-19.9]	0.028ˆ*

*Data are medians [25th–75th quartile]. ^∗^Significant difference between pre- and post-operative scans (p < 0.05). MRI, magnetic resonance imaging; TLV, total lung volume; T_*1*_ – VIBE: T_*1*_ – weighted gradient-echo volumetric interpolated breath-hold examination.*

## Discussion

The main findings of our study are that: (1) in an isolated *ex vivo* lung model, MRI signal intensity could be expressed as a quadratic function of lung density; (2) normalization of lung MRI signal to a reference signal, namely muscle or water, was required; (3) MRI signal intensity decreased linearly as gas content increased; in patients, (4) the dorsal versus ventral lung regions had a higher MRI signal intensity also pre-operatively; and (5) post-operative scans had higher signal intensity and higher amounts of non-aerated and poorly aerated tissue.

To our knowledge, this is the first study investigating the value of standard MRI sequences, as intended for clinical use, to provide an estimate of lung aeration without the use of dedicated research scanners, lung-specific MRI sequences or gaseous contrast media. This could represent a progress in the development of clinically feasible MRI protocols to assess lung aeration, without requiring ionizing radiations, which is a rapidly developing field of research (Ball et al., 2017b).

### *Ex vivo* Model

Our main aim was to test the hypothesis that the intensity of the MRI signal was a function of the degree of inflation of the lung, thus its density. For this purpose, we first used an *ex vivo* isolated lung model, as previously described in a study validating quantitative lung ultrasound analysis (Corradi et al., 2013). We instilled a fixed amount of hypotonic saline (0.5 ml per gram of lung tissue), as we observed in our previous study that higher amounts did not result in a relevant increase in the amount of retained EVLW. While affected by several limitations, such as the lack of perfusion and the absence of the chest wall attenuating lung signal, this model allowed us to have control on the degree of inflation of the lung. Moreover, in a restricted number of lung samples we instilled hypotonic saline as an attempt to control for the potential role of edema in modifying the relation between lung aeration and MRI signal intensity. In fact, in T_2_ – HASTE sequences, where static fluids have an extremely high signal intensity, we observed a weaker correlation, heavily affected by between-samples differences, including the presence of instilled fluids, unlike in T_1_ – VIBE scans (see **Figure 1** and **Table 2**).

### Image Acquisition

We used a 1.5 T scanner and the mentioned sequences, as these were already used to acquire lung images for research purposes in our institution. MRI of the lung is hampered by several factors: multiple interfaces between tissue, with diamagnetic properties, and air, which has an extremely low proton density and contains gaseous paramagnetic oxygen (Mulkern et al., 2014). This generates a highly inhomogeneous local magnetic field gradient, with clusters of signal much smaller than the conventional voxel size. The presence of such gradient implies a fast signal decay, that can be investigated on a 1.5 T scanner using gradient-echo sequences, with or without respiratory gating (Wild et al., 2012). Low-field MRI is less sensitive to signal inhomogeneity but has the disadvantage to generate noisy images that require averaging to recover the SNR. In fact, especially in patient scans, we obtained low-quality images that would have probably been inadequate for conventional clinical evaluation (Biederer et al., 2012). However, we aimed at achieving a degree of precision adequate for aeration estimation, rather than for anatomical assessment. In both experimental and clinical scans, we could perform segmentation and demonstrated a relationship between lung aeration and MRI signal intensity. This relationship is explained by the fact that proton density per voxel is determined by lung gas content: changes in lung volume of equal magnitude will cause corresponding changes in proton density, with corresponding modifications in MRI signal intensity (Bankier et al., 2004).

### Image Analysis

The lack of a standard calibration of gray units in MRI imaging represented a major challenge for our study. From the *ex vivo* model, we concluded that a normalization to a reference signal was necessary, and we opted for muscle as it is constantly found in clinical MRI of the thorax and easily definable. Normalization of MRI to a reference organ has been already proposed in other contexts to allow semi-quantitative analysis, (Moshiri et al., 2013) but its efficacy is affected by the between-subjects differences in the reference organ signal. The MRI signal of muscles could be influenced by several factors such as nutritional status, hydration, and age (Carlier et al., 2016). We limited our analysis to non-aerated and poorly aerated compartments of the lung, since the density of hyper-aerated tissue can be as low as 0.05 g/mL, (Ball et al., 2016), leading to an unacceptably low SNR.

### Translation to Humans

Magnetic resonance imaging signal attenuation compared to deflated lung samples was linearly and inversely correlated with the increase of gas content. This analysis was crucial to overcome the intrinsic limitations of the *ex vivo* model, and to allow translation to patients’ scans. First, we could not directly translate the lung MRI – density relationship from the model to patients, as the *ex vivo* lungs were non-perfused and not surrounded by chest wall. The latter difference, while of limited relevance for CT image reconstruction algorithms, is of crucial importance in MRI, where the weak signal arising from low-density areas of the lung must pass through the chest wall to be detected by the receiving coil (Wild et al., 2012). This likely explains the difference between MRI signal intensity in *ex vivo* lungs (**Figure 2**) and patients (**Figure 5**). Also, it is the probable reason why we could not simply use the quadratic function shown in **Figure 1**. Conversely, the relationship illustrated in **Figure 4** refers to relative changes from a deflation status. Thresholds were then translated measuring the average signal intensity of the central part of lung atelectasis, by multiplying the attenuation thresholds of the *ex vivo* model and the atelectasis signal.

### Possible Implications

This study has several potential applications. We demonstrated that fast MRI sequences available on most scanners, as intended for clinical use, can provide an estimation of lung aeration. Compared to bedside techniques such as lung ultrasound and electric impedance tomography it has the advantage of a higher spatial resolution and the possibility to map the whole lung aeration. Compared to CT, the technique is radiation-free, and could deliver information in conditions were CT was not used to limit radiation exposure, such as infant respiratory distress syndrome, or where the risk benefit ratio would not justify CT, for example in peri-operative medicine, or when investigating experimental ventilation modes. However, compared to CT, MRI has a lower spatial resolution, a less favorable SNR and requires longer acquisition time. This would probably imply that further technical improvements are required to translate our findings in a wider application field, e.g., in the pediatric setting, where the extremely elevated respiratory rate would require sampling smaller volumes or the use of respiratory gating. However, MRI has several pitfalls related to the necessity of moving the patient to the MRI scanner, a longer acquisition time making the technique more sensitive to motion artifacts, and the need for specific equipment compatible with MRI.

### Limitations

This study has several limitations. First, the *ex vivo* model was non-perfused and isolated, and no comparison was performed with CT or other imaging techniques. However, this method allowed us to control lung size in a wider range compared to an animal study, limiting the interferences from the chest wall. Moreover, we could validate quantitative MRI analysis versus gravimetry, thus precisely estimating density at different inflation levels. Second, we could not compare patient scans with a reference imaging technique, such as CT, as it would have been ethically unacceptable. In the future, validation studies should consider using low-dose CT acquisition protocols (Chiumello et al., 2014; Ball et al., 2017a,b) to validate quantitative MRI. Third, in patient scans the effect of the chest wall could have been different between patients, however, the study excluded obese subjects yielding a rather homogeneous chest wall thickness. Fourth, the muscle reference could have been affected by rest, fluid balance, age and other unknown factors. Nonetheless, we interpreted the low variability in lung-to-muscle ration in pre-operative scans as an indirect proof in favor of such normalization.

## Conclusion

Quantitative MRI assessment of aeration required normalization of the signal to a reference, either water or muscle. MRI signal intensity in T1 – VIBE scans could be expressed as a quadratic function of density in our *ex vivo* model. MRI intensity decreased linearly at increasing gas content. In patients’ scans, quantitative MRI could both identify the ventro-dorsal gravitational aeration gradient, and the loss of aeration in the post-operative period. According to these findings, MRI performed with clinically available sequences seems to be a promising tool to assess lung aeration. Further validation and clinical trials are warranted to assess the potential applications of quantitative MRI.

## Author Contributions

LB, PP, and MGdA designed the study. LB, AB, PS, MH, and VH collected the data. LB and VH analyzed the data. LB, PP, KC, PS, NP, MS, and MGdA interpreted the results and wrote the manuscript. LB and AB are equally contributing first authors. MGdA and PP share senior authorship. All the authors revised the final version of the manuscript.

## Conflict of Interest Statement

The authors declare that the research was conducted in the absence of any commercial or financial relationships that could be construed as a potential conflict of interest.

## Footnotes

1 www.itksnap.org

2 www.r-project.org
