# Supplementary material for: Magnetic Resonance Imaging for Quantitative Assessment of Lung Aeration: A Pilot Translational Study
Source: Front Physiol. 2018 Aug 13;9:1120. doi: 10.3389/fphys.2018.01120 (PMC6099446; doi:10.3389/fphys.2018.01120)
Supplement: Supplementary file 1 [file Image_1.pdf]

Magnetic Resonance Imaging for Quantitative Assessment of Lung Aeration: a Translational Study

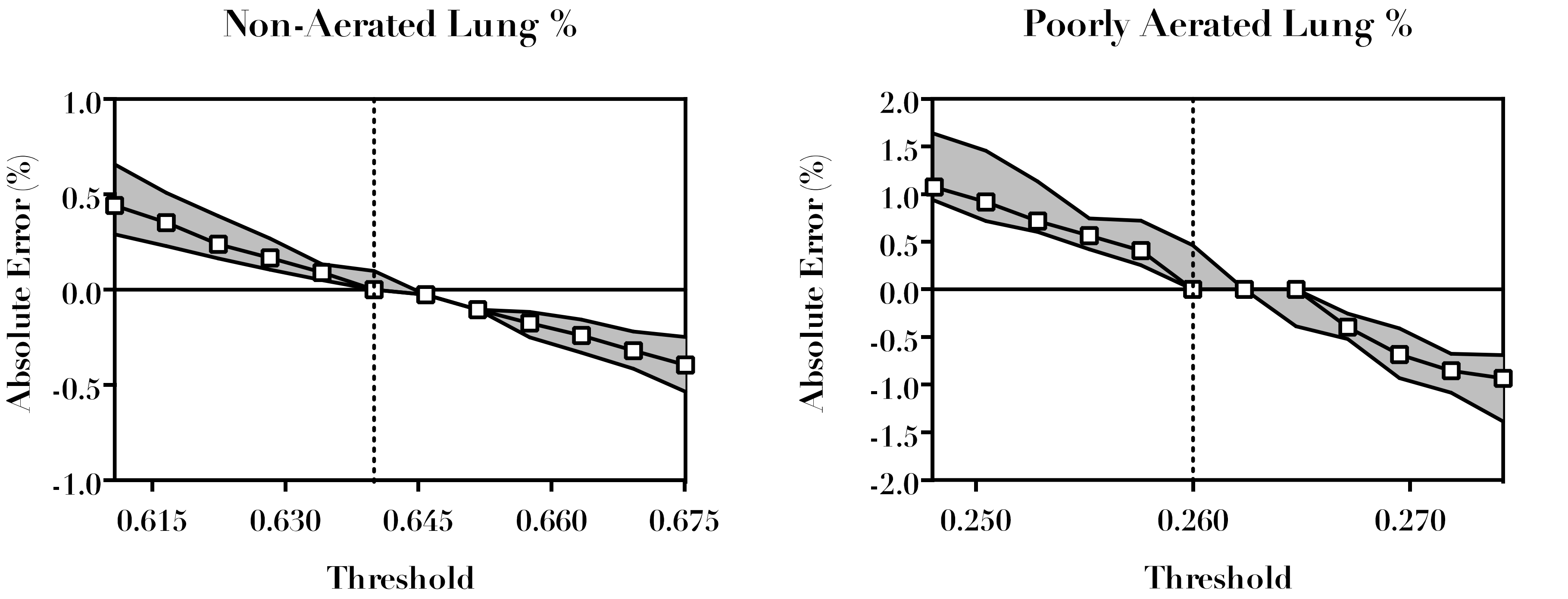

Supplemental figure: Robustness of in-vivo MRI thresholds. This figure illustrates the absolute error in the estimation of non-aerated (left) and poorly aerated (right) compartments introduced by a variation in MRI attenuation thresholds by ± 5 %.
